# Routes for Metallization of Perovskite Solar Cells

**DOI:** 10.3390/ma15062254

**Published:** 2022-03-18

**Authors:** Janusz Edward Jacak, Witold Aleksander Jacak

**Affiliations:** Department of Quantum Technologies, Wrocław University of Science and Technology, Wyb. Wyspiańskiego 27, 50-370 Wrocław, Poland; witold.aleksander.jacak@pwr.edu.pl

**Keywords:** perovskite solar cells, Shockley–Queisser limit, metallization of solar cells, multishell nanoparticles, prolate nanoparticles

## Abstract

The application of metallic nanoparticles leads to an increase in the efficiency of solar cells due to the plasmonic effect. We explore various scenarios of the related mechanism in the case of metallized perovskite solar cells, which operate as hybrid chemical cells without p-n junctions, in contrast to conventional cells such as Si, CIGS or thin-layer semiconductor cells. The role of metallic nano-components in perovskite cells is different than in the case of p-n junction solar cells and, in addition, the large forbidden gap and a large effective masses of carriers in the perovskite require different parameters for the metallic nanoparticles than those used in p-n junction cells in order to obtain the increase in efficiency. We discuss the possibility of activating the very poor optical plasmonic photovoltaic effect in perovskite cells via a change in the chemical composition of the perovskite and through special tailoring of metallic admixtures. Here we show that it is possible to increase the absorption of photons (optical plasmonic effect) and simultaneously to decrease the binding energy of excitons (related to the inner electrical plasmonic effect, which is dominant in perovskite cells) in appropriately designed perovskite structures with multishell elongated metallic nanoparticles to achieve an increase in efficiency by means of metallization, which is not accessible in conventional p-n junction cells. We discuss different methods for the metallization of perovskite cells against the background of a review of various attempts to surpass the Shockley–Queisser limit for solar cell efficiency, especially in the case of the perovskite cell family.

## 1. Introduction

The contribution of installed photovoltaics (PVs) to the global electricity demand reached more than 2.7% in 2020, i.e., more than 750 GW (4.3% in the EU) [1] and this figure continues to grow. PVs, together with wind and hydro-power, are systematically increasing the energy supply on the global scale. The International Energy Agency predicted a growth in the use of PVs by 900 GW by 2024 [2].Though the cost of PV energy production is still larger than that of coal or gas on average, in some instances, PVs offer the cheapest source of electrical power in regions with high sunlight exposure, such as in Qatar, with a price as low as 0.015 USD/kWh [3]. The intensive development of PV cell production will, however, reduce the costs of PV installation and increase their profitability in other places that are well exposed to sunlight, especially in Africa, Australia and, on the other hand, in leading energy consumers such as the EU, USA, China and India. It is thus very important to optimize the efficiency of solar cells.

The most popular Si cells achieve an efficiency of 26.7% (mono-crystalline laboratory set) and of 24.4% for (multi-crystalline Si wafers). Furthermore, other material cells, using thin-film technology, offer growing efficiencies up to 23.4% for CIGS and 21.0% for CdTe solar cells. Constructions using multi-junction solar cells achieve an efficiency up to 47.1% today. However, thin-film or multi-junction solutions are usually more expensive and the production of Si cells is dominant for mass applications (with a 95% share). In 2020, cell production in Asia accounted for 95% of the global Si PV module production (mainland China supplied a share of 67%). The share of Si mono-crystalline technology is dominant here, representing 84% of total Si cell production in 2020 (compared to 66% in 2019). Simultaneously, a PV market shift from a subsidy-driven model to a competitive cost model is observable in all countries, which additionally motivates efforts to increase cell efficiency and to lower production costs.

The durability time-span of Si-type cells ranges from 20–30 years. However, the processes involved in their recycling and production are complicated and involve energy costs. Therefore, new, more easy and environment-friendly technologies are desirable. Third-generation chemical solar cells are easier to produce and recycle, but their efficiency is much lower than that of Si-based cells. Chemical cells operate in a manner distinct from semiconductor cells with p-n junctions and their production involves low temperatures and low energy consumption. This class also includes hybrid chemical cells, in which the photon-absorber is a semiconductor but the separation of electric current carriers is similar to that found in conventional dye chemical cells at the interface with the electron or hole transport layer. The development of hybrid chemical cells resulted in the invention of perovskite solar cells with ultra-thin solid absorber–semiconductor layers of perovskites deposited on the substrate through a simple chemical process from liquid precursors at normal conditions, even via ink-jet or screen printing.

Perovskite solar cells have arisen in the past few years (compared to 2009, when the prototype of a perovskite cell with an efficiency of 3.8% was demonstrated [4]) as a very promising candidate for large-scale commercial use. Their efficiency has now surpassed 25%—South Korea’s Ulsan National Institute of Science and Technology (UNIST) and the Swiss Federal Institute of Technology Lausanne (EPFL) announced in April 2021 a record efficiency of 25.6% (for a single-junction perovskite cell), which edged out the previous record of 25.2% (demonstrated by the Massachusetts Institute of Technology) [5,6]. Such high efficiencies of perovskite solar cells make them competitive with Si solar cells, which are the most popular at present. The commercial production of fully printed perovskite cells on thin, flexible foils was commenced in 2021 by Saule Technologies [7], offering flexible and low-weight systems (10 times lighter than traditional silicon PV installations), with the possibility of printing in various shapes, patterns and colors.

Perovskite cells can be produced via low-cost chemical methods at temperatures below 100 ^o^C, allowing roll-to-roll printing technology using liquid precursors [8]. The ultimate ultra-thin multilayer cells can be deposited on various substrates, including plastic stripes, glass, textiles or even the ceramic materials used for building construction elements, such as walls and roofs. The main drawback of these new universal and flexible solar systems is their relatively low durability. They can be degraded by atmospheric oxygen and water; thus, their encapsulation in plastic or glass is necessary. However, the application of additional protective layers increases costs and complicates the production process. To avoid these obstacles, some alternate methods are being intensively researched at present with the aim of increasing the durability of perovskite cells through the modification of the chemical composition of the precursors and through some special physical treatment techniques (such as surface modification using an ion beam) [9,10]. Another inconvenient factor hampering the use of perovskite cells is the composition of perovskites, which can include hazardous lead, which would pollute the environment. To remedy this danger, researchers have explored lead-free replacement materials with similar chemical and electronic structures. However, such solutions result in the lowering of the cells’ efficiency [11,12,13].

It has been experimentally demonstrated that through the application of metallic nanoparticles, one can additionally increase the efficiency of perovskite solar cells [14,15,16]. In [16], the highest relative increase (of 40%) in total efficiency was achieved through the application of rod-shapes gold nanoparticles incorporated into the crystalline perovskite layer close to the interface with the charge transporter. The elongated shape of applied metallic admixtures causes the splitting of plasmonic resonance, which improves the perovskite’s susceptibility to plasmon radiation.A lower but still pronounced efficiency increase was demonstrated in other experiments utilizing spherical but multishell nanoparticles, composed of gold with thin coating of SiO${}_{2}$ [14], and complexes with silver cores coated with TiO${}_{2}$ and a top layer of benzoic acid usingfullerene C${}_{60}$ [15]. Metallic cores become active when plasmons are excited by photons from sunlight, and through near-field zone coupling of plasmons to band electrons in the perovskite layer, plasmons mediate the creation of excitons in the semiconductor absorber. Outer coverings of the metallic core, consisting of a thin dielectric or organic layer, are used to minimize the morphology perturbations in the multilayer perovskite cell [14,15].

Although the metallization of solar cells is also beneficial in conventional p-n junction solar cells, such as Si cells (for a detailed presentation of this method, cf., [17], Chapter 8), the deposition of metallic nanoparticles on the top of crystalline p-n junction cells requires the application of additional covering layers in order to fix and protect surface admixtures. Such coverings increase the cell production costs, and the metallization of Si cells is not a popular area of research at present, though it could enhance efficiency by 10% on average (cf., the comparison of various experimental setups provided in [17], Table 8.3). This problem disappears in the case of perovskite cells, in which metallic nanoparticles can be located inside the absorber, embedded in the perovskite or at the interface between the absorber and the charge transporter simply through the use of liquid precursors doped with metallic nanoparticles during the process of ink-jet printing. The mass of Au required for the typical $10^{8}$/cm${}^{2}$ surface concentration of particles with a radius of 40 nm is only $5\times 10^{-3}$ g/m${}^{2}$, which equates to a low material cost of ca. 0.30 USD per 1 m${}^{2}$ in the case of Si-metallizedcells. For the metallization of perovskite cells, a similarly small amount of metal is needed.

In the present paper we focus on the plasmonic PV effect, examining various factors which can optimize this effect in the new generation of perovskite solar cells. In metallized perovskite solar cells [14,15,16], the mechanism of the plasmon-aided photovoltaic effect is different than that observed in p-n junction devices covered with metallic nanoparticles [17,18,19,20,21,22,23,24,25,26,27]. Perovskite cells operate in a different manner to p-n junction cells (for a recent review of perovskite cells, see [6]). In p-n junction cells (e.g., Si cells, CiGS or GaAs-based cells) in the depletion region near the junction of the p-doped semiconductor layer and the n-doped one, the dissociation of photo-induced excitons takes place. The voltage (multiplied by the electron charge) of the junction barrier is typically one order of magnitude greater than the binding energy of the excitons (e.g., in p-n Si this voltage is of the order of 1 V, whereas the binding energy of photo-excitons is lower than 100 meV); thus, the dissociation of excitons is almost an instant process, independent of the variation of the exciton binding energy. In perovskite cells, the dissociation of photo-excitons takes place at the interface with the electron (or hole) transport layer due to the gradient of the edges of energy bands across the interface [6]. The lower the binding energy of the excitons, the more rapidly dissociation takes place. Plasmons from metallic nanoparticles can greatly accelerate this dissociation [14] as they can reduce the binding energy of the excitons [28,29].

In p-n junction cells, metallic components mediate and strengthen the absorption of photons and this effect has an optical character. In perovskite cells, a strong plasmonic effect is related with an intrinsic electrical mechanism via the acceleration of exciton dissociation. The latter effect is not possible to induce in p-n junction cells; however, in perovskite cells both optical and electrical effects are possible in principle, though they require the application of nanoparticles with different plasmonic characteristics.

In the present paper, we explore in detail and compare various alternative routes for the use of plasmonic components, which may be beneficial for the improvement of perovskite solar cell efficiency, while also utilizing the optical absorption plasmonic effect in addition to the inner electrical one. The main goal is to activate, through the special tailoring of metallic components, the optical absorption channel of the plasmonic photovoltaic effect in perovskite cells, which is very poor in these cells due to the large gap and large masses of carriers in CH${}_{3}$NH${}_{3}$PbI${}_{3}$ [28]. We demonstrate herein that through the application of metallic nanocomponents with blue-shifted or split plasmonic resonance, using multilayer core-shell multi-metal structures and metallic nanoparticles with an elongated shape, it is possible in perovskite solar cells to exploit both channels of the plasmonic PV effect simultaneously—both the optical and electrical channels—which allows us to surpass the efficiency increases observed for each of these channels separately.

The paper is organized as follows. In the next paragraph we briefly review the factors which limit the efficiency of solar cells, conventionally expressed as the Shockley–Queisser efficiency limit. A review of methods aiming to approach or surpass this limit is then presented, especially with regard to the perovskite solar cell family, with varying chemical compositions used for the absorber. In the subsequent section, we briefly present a description of the theoretical model we used to assess the plasmonic photovoltaic effect. This model has been formulated previously [17], so we refer herein to these prior publications and omit formal details and repeated information. Using this quantum model, the optimization of the plasmonic effect in perovskite cells (with varying chemical compositions used for the absorber) is analyzed in the subsequent paragraph. In this final discussion, we prove that via splitting of the plasmon resonance in elongated metallic nanoparticles and/or utilizing multi-shell double-metal particles, it is possible in perovskite cells to take advantage of both optical (absorption) and electrical inner plasmonic effects simultaneously in perovskite cells, achieving an increase in their efficiency beyond the limits of p-n junction cells (in which only the optical channel is possible).

## 2. Shockley–Queisser Efficiency Limit

The theoretical estimation of the upper limit for efficiency of solar cells is known as the Shockley–Queisser efficiency limit, also known as the detailed balance limit [30]. This limit holds only for idealized p-n junction cells and accounts for losses induced by the thermal irradiation of a cell, the recombination of electron and hole carriers and sunlight spectrum losses beneath and beyond the band-gap of semiconductors, assuming that photo-excitons are created by incident photons mostly on the p-side of the junction. These factors cause a competing dependence of the V–I efficiency of the cell exposed to sunlight with respect to the semiconductor bandgap, which results in a maximum of ca. 33.7% for the overall efficiency at the bandgap ca. 1.34 eV (which is slightly larger than in Si, where the gap is ca. 1.14 eV) [31].

Nevertheless, many other effects which reduce solar cell efficiency are not taken into account in the Shockley–Queisser efficiency limit estimation. Examples of these include the nonradiative recombination of excitons, including mediation by phonons, admixtures, defects or other carries along the Auger recombination scheme. On the other hand, various methods to surpass the Shockley–Queisser efficiency limit have been proposed and are still in development. Such a methods consist of concentrations of illumination using mirrors or lenses, the application of quantum dots to better utilize energy excess beyond the bandgap (assumed to be lost due to the thermalization involved in the conventional Shockley–Queisser limit derivation), producing a mid-energy state within the gap, photon up-conversion or two-photon-absorption for energies lower than the gap, multi-exciton generation, fluorescent downshifting and tandem or multilayer cell architectures. Metallization at the nanoscale is also one of these methods.

The Shockley–Queisser efficiency limit is evaluated for p-n junction cells exclusively, whereas perovskite cells have no p-n junction and e-h pairs are excited throughout the whole perovskite layer in contrast to p-n junction cells, in which electrons are assumed to interact with photons only on the p-side of the junction. Because of the different locations and mechanisms of exciton creation and dissociation in chemical cells in comparison to p-n junction cells, the recombination of excitons during the diffusion to the interface with the charge transport layer, their mobility and the mobility of liberated charge carriers on their way back to the reciprocal electrodecontribute to the overall efficiency of perovskite cells in an altogether different manner than in conventional p-n junction cells. Therefore, the Shockley–Queisser efficiency limit (of ca. 33% for an optimal bandgap) ought to be considered approximate and dubious for perovskite cells. The same can be stated abouttheoretical estimations of the efficiency increase due to tandem (and multilayer in general) cell architectures.

## 3. Ways to Approach or Surpass the Shockley–Queisser Limit in Perovskite Cells

**Figure 1:**
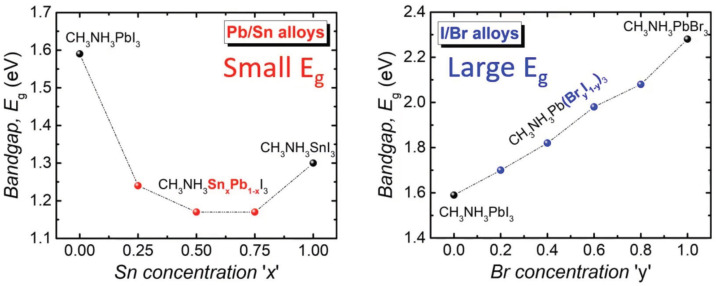
Variation of the forbidden gap in perovskites versus the chemical composition. There are two families of perovskites, with low (**left**) and high (**right**) gaps, which can be utilized to construct perovskite-perovskite tandem cells or to optimize the plasmonic effect [34].

In perovskite cells, attempts to reduce nonradiative photo-exciton recombination both in bulk and at the interface with the hole or electron transport layers have been undertaken [32] with the aim of increasing efficiency and surpassing the Shockley–Queisser efficiency limit despite its approximate character, as this limit refers to a case in which the recombination of electron–hole pairs is assumed to be only radiative in a single-bandgap semiconductor p-n junction cell. The specific scheme of operation of chemical cells including perovskite ones makes the recombination of excitons a much more important factor than it is in p-n junction cells, because in these cells e-h pairs are excited throughout the whole semiconductor layer in contrast to p-n junction cells, in which electrons are photoactive mostly on the p-side of the junction. In perovskite cells, nonradiative recombination is also important at the interface with the large-gap semiconductor (the electron or hole transport layer) according to the Shockley–Read–Hall scheme. In [32], the authors studied ways of overcoming this obstacle. The reduction of nonradiative recombination in the perovskite layer is explored via changing the perovskite composition, the application of some additives, structural engineering of grain orientation and size, the modification of the precursor solution, and innovative post-treatments. In particular, increasing grain size, reducing passivation defects and improving carrier transport and separation were found to be promising in regard to approach the limits of efficiency. Tailoring of the large and small band-gap via composition variations—see Figure 1—allows one to design perovskite-perovskite tandem cells which are better accommodated to the sunlight spectrum and which exhibit a strongly increased open-circuit voltage [33,34,35,36]. The direct application of the conventional Shockley–Queisser estimation method to a doubled-cell structure with a large bandgap p-n junction component beyond the lower bandgap p-n junction results in 42% efficiency for such an optimal tandem system. It has been reported that in perovskite-perovskite large-small gap tandem systems, ca. 80% of this limit is achieved without any involvement of the p-n junction [34].

To approach or even surpass the Shockley–Queisser efficiency limit through the application of metallic nano-components, plasmonic components in the form of several-nanometer- to several-tens-of-nanometer-sized metallic particles, sparsely distributed inside the optically active medium, have been used. These can mediate the absorption of photons and induce some modifications in the internal electricity of the cell.

The plasmonic PV effect is known from observations in conventional p-n cells, in which low-density surface coverings with metallic nanoparticles (Au, Ag or Cu with diameters of several tens of nanometers) significantly strengthened the absorption of photons. A review of experimental achievements in Si-based photovoltaic devices metallized at the nanoscale is presented, e.g., in [17], Chapter 8. THe plasmonic effect consists (in p-n junction cells) of the strong coupling of surface plasmons in metallic nanoparticles with band electrons in the substrate semiconductor in the near-field zone of plasmon radiation. This coupling opens up a very efficient channel to transport energy from plasmons to electrons. The latter are excited (on the p-side of the p-n junction) from the valence band to the conduction band. In addition to leaving behind holes in the valence bands, they form excitons with binding energies of the order of a few tens of meV. If the creation of excitons takes place in the depletion region of the p-n junction, they are instantly dissociated by the junction voltage (which is ten times larger than the binding energy per electron charge) into free-moving electrons and holes, which are next spatially separated by the same voltage and which tend towards the opposite electrodes of the cell. If excitons are created in a more distant region of p-side of the junction, they must diffuse toward the barrier voltage. On the way, they can recombine radiatively or non-radiatively, which causes losses in overall efficiency.

In perovskite cells, the dissociation of excitons has a more subtle effect at the sharp slope of the edges of conduction bands at the interface of the perovskite layer with the electron transport layer. The latter is usually a large-gap semiconductor such as TiO${}_{2}$ or Si${}_{2}$O${}_{3}$. The gradient of the band energy edges disrupts excitons and directs electrons into the electron transport layer. Liberated holes diffuse towards the opposite electrode. In the inverted architecture of a perovskite solar cell, with a hole transport layer instead of an electron one, the scheme of the operation is similar. In this case, holes are extracted first at the interface with another material, which is the hole transporter. It is clear that in perovskite cells the binding energy of excitons is crucial for the efficiency and velocity of the charge extraction, which is completely opposite to the case in p-n junction cells, where the binding energy of excitons has a rather marginal significance, connected only with the exciton recombination rate (the higher the exciton binding energy is, the more rapidly recombination takes place). In perovskite cells, the reduction of the binding energy in excitons may strongly influence the cell’s efficiency, eventually increasing the photocurrent. This has been observed experimentally [14].

It has been proven both experimentally [14] and confirmed theoretically [29] that in perovskite cells, spherical metallic nanoparticles of Au do not cause a strengthening of the light absorption, although the same size and concentration of Au particles strongly increase absorption (even by a factor of two) in Si p-n junction cells [18]. This passive optical channel of the plasmon PV effect in perovskite cells is caused by the large masses of electrons and holes and the relatively large bandgap in the perovskite absorber.

Despite the poor absorption enhancement induced by metallic admixtures, experiments have indicated a relatively strong enhancement of the overall efficiency of perovskite cells due to metallization [14,15,16]. The strong overall plasmonic effect in perovskite cells, even up to a 40% relative increase in the efficiency, is related with an intrinsic electrical mechanism, not an optical one, which is impossible to attain in p-n junction cells. This effect in cells operating according to a chemical scheme consists in a reduction of the binding energy of excitons by plasmons, which eventually accelerates the dissociation of excitons at the interface with the electron (or hole) transport layer. The reduction of the exciton binding energy is the strict quantum effect caused by indirect interband transitions in the perovskite when electron-hole pairs are excited by plasmons in their near-field radiation zone. This near-field radiation of surface plasmons, oscillating in metallic nanoparticles embedded in the perovskite layer (usually close to the interface with the electron or hole transport layer) is a perturbation that is not translationally invariantand which therefore induces indirect interband hopping of electrons. This is not constrained by the momentum conservation rule, in contrast to the ordinary photo-effect, in which only direct interband excitations, conserving momentum, were affected, as a result of translational invariance.The increase in the probability of indirect interband electron transitions without the conservation of momentum (pseudomomentum in the Brillouin zone) is high and causes a large number of plasmon-mediated excitons which have a nonzero relative momentum of their components, electrons and holes.Oppositely oriented momenta (pseudomementa in fact) of both carriers tend to pull apart the electron–hole pair, which strongly reduces its binding energy (by more than 50%, as evidenced experimentally [14]).

The similar effect occurring in p-n junction cells (such as Si-based cells or CIGS) is not important because the junction voltage usually greatly exceeds the binding energy of photo-excitons and their dissociation is almost instant in the region of depletion, regardless of the variation in the exciton binding energy. Thus, in p-n junction cells, the reduction of the binding energy of excitons created via the mediation of plasmons does not play a role in cell operation. In chemical cells, the scheme of operation is different and excitons diffuse first to the interface with the electron or (hole) transporter, where due to the gradient of the band edges across the junction, electron–hole pairs decouple, and this process can be significantly accelerated by lowering the exciton binding energy.

An essential difference in U-I cell characteristics has been observed due to the plasmonic PV effect in chemical hybrid perovskite cells in comparison to the plasmon-mediated efficiency increase in metallized Si-cells or other semiconductor cells operating in the p-n junction diode fashion. In the latter case, the plasmon-induced absorption dominates the overall effect, whereas in the case of perovskite cells the internal electrical plasmon-effect plays a more important role. This difference is noticeable in the V–I characteristics of metallized cells. An increase in the absorption of incident photons from sunlight via plasmon nanoparticles in the case of p-n junction cells causes an increase in the open-circuit voltage. In chemical perovskite cells, metallization induces an electric effect, which manifests itself on the other hand in an increase in the photocurrent.

**Figure 2:**
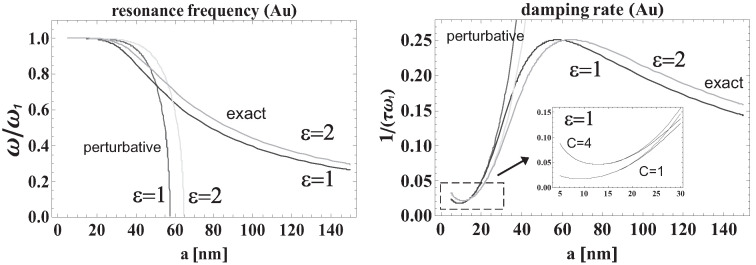
Resonant frequency of the dipole mode of surface plasmons versus the metallic (Au) nanoparticle radius (**left** panel). Damping rate of dipole plasmon mode versus nanoparticle radius (**right** panel)—the maximum level of damping at the “plasmonic size” of a nanoparticle radius of ca. 50 nm for Au, is considered to be causedby giant Lorentz friction at this scale of spatial confinement. The resonant frequency and the damping rate of dipole surface plasmons (visualized in the figure) indicate that plasmons are not harmonic oscillators; they oscillate even under extremely strong damping without an over-damped regime, which terminates harmonic oscillations (as shown in the figure as perturbation solutions in contrast to exact ones, including Lorentz fiction). For comparisons, two situations are presented—Au nanoparticles in a vacuum (ε=1) and in dielectric surroundings with other values of dielectric susceptibility.

Incident photons from sunlight are absorbed in metallic nanoparticles due to the extremely large absorption rate of surface plasmons at the nanometer-scale confinement of the metallic system.It was proven in [17], Chapter 5, that the absorption of electron plasma that is spatially confined to the nanoscale size is much larger in comparison to bulk systems and ultra-small metallic clusters, because of the predominant role of the radiative Lorentz friction of plasmons with a maximum diameter of nanoparticles (for Au particles in a vacuum) of ca. 100 nm. The mirror symmetry of light absorption and emission [37] causes strongly radiating plasmons to strongly absorb radiation as well. Metallic nanoparticles thus act as highly efficient collectors of sunlight energy in their surface plasmon modes, and even at small concentrations, they can capture a large fraction of incident photons. This energy is quickly radiated due to the large Lorentz friction of plasmons at the nanometer scale of spatial confinement—cf. Figure 2, where the resonance frequency (or wavelength) and damping of dipole surface plasmons in an Au nanosphere is presented versus its radius. Nevertheless, when metallic nanoparticles are embedded in a semiconductor, the energy collected in plasmons is much more quickly transferred to the band electrons of the semiconductor. This channel is faster than the Lorentz friction and can be estimated by means of the Fermi golden rule applied to the quantum transition induced by coupling of electrons to plasmons within their near-field zone of radiation [38,39]. The time rate for the energy transfer from plasmons to nearby electrons is much shorter than for other plasmon damping channels [17].

The spatial range of the plasmon-electron coupling has been checked experimentally and it occurred of the micrometer scale [27], which exceeds the surface-plasmon resonance wavelength (typically of order of 0.5 μm for Au, Ag or Cu nanoparticles). The finite spatial range of plasmon-electron coupling causes, however, that the n-p junction in conventional solar cells must be located within such distance from nanoparticles to optimize creation of excitons by surface plasmons. In perovskite solar cells the optimal thickness of optically active layer is of order of 300 nm [40,41], thus the whole semiconductor volume is conveniently inside the range of plasmonic effect at arbitrary location of metallic nanoparticles. This is a great advantage of metallization of perovskite cells in comparison to p-n junction cells.

## 4. Theoretical Assessment of the Efficiency of Coupling between Plasmon Dipoles in Metallic Nanoparticles and the Perovskite Semiconductor

In this section we refer to the theoretical model derived previously by the authors to describe the surface plasmon-mediated photovoltaic effect in solar cells (in p-n junction solar cells, such as nano-metallized Si-type cells [17] and in metallized perovskite cells [28]). These theoretical models are quantum and microscopic in order to account for the plasmonic effect via the application of the Fermi golden rule to the coupling of surface plasmons in metallic nanoparticles to band electrons in a nearby semiconductor. This is distinct from the classical consideration of the plasmonic effect provided in the solution of the Maxwell–Fresnel problem and the determination of the strengthening of the electrical field of the incident photon wave-function near the curvature of metallic nanoparticles. This strengthening increases the photon absorption in the adjacent semiconductor, but surprisingly accounts for only 5% of the total plasmonic photovoltaic effect. The rest of absorption increases via plasmons, i.e., 95% of the total observable absorption strengthening is caused by the quantum effect derived in the abovementioned studies. However, in the case of perovskite cells, a new channel of the plasmonic effect has been discovered [29], which is also purely quantum and cannot be accounted for classically—it is of an electrical, not an optical (absorption), character.

In the conventional photo-effect [42] the interband transitions induced by photons are confined only to vertical ones between electron states in the valence and conduction bands, i.e., between electron states with almost the same momentum (pseudomomentum in the Brillouin zone). This is due to the conservation of momentum and the negligibly small momentum of sunlight photons. If the energy of a photon is accommodated into the forbidden gap of the semiconductor, then its momentum, $q=\frac{E_{g}}{c}$ ($E_{g}$m is the gap of the semiconductor; *c* is the velocity of light), is very small, which is a consequence of the very large velocity of light, *c*. When incident sunlight photons first excite plasmons in metallic nanoparticles (mostly in the surface plasmon dipole mode in nanoparticles of several dozen nanometers in size, for which the dipole approximation regime holds), then the interaction of localized plasmons with band electrons in the surrounding semiconductor is not translationally invariant, which removes the momentum conservation constraints for interband transitions of electrons induced by plasmons in the near-field zone of plasmon radiation. The resulting non-vertical interband transitions of electrons contribute to the photo-effect, which enhances the overall cell efficiency.

**Figure 3:**
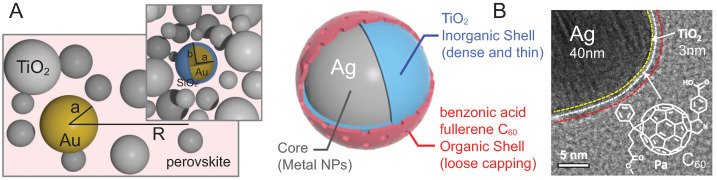
(**A**). Spherical Au nanoparticles (with the radius *a*) are capable of being embedded in the perovskite layer due to the crystallization of this layer using liquid precursors with an admixture of metallic nanoparticles. Conveniently, nanoparticles of gold can be located close to porous basis of TiO${}_{2}$ or (Al${}_{2}$O${}_{3}$) of the electron transport layer. The density of particles is low, of the order of 1 wt% with respect to the porous basis of TiO${}_{2}$ (as in the experiments reported in [14]). Such a low concentration of Au nanoparticles does not disturb the morphology of the cell layers. Moreover, they are additionally coated with a dielectric layer of SiO${}_{2}$ up to a final radius of b≃48 nm (bare nanoparticles have a radius of a≃40 nm) in order to reduce contact with the naked metal and related structural distortions. (**B**). Multi-shell plasmonic particle Ag@TiO${}_{2}$@organic-shell (left) and its HRTEM image (right). The organic outer-coating layer with the chemical structure depicted in the right picture is applied to reduce the aggregation of plasmonic particles. Such multi-shell Ag particles were used in the experiments reported in [15] in an inverted-architecture perovskite cell with a hole–absorber PIDTT layer to which the organic coating of Ag multi-shell particles was specially matched.

This is a quantum effect, which can be described in terms of quantum transitions within the scheme of the Fermi golden rule [37]. According to this rule, the probability of the inter-band transitions per time unit is proportional to the square of the matrix element of the perturbation potential between the initial and final states of electrons and to the Dirac delta function assuring the conservation of energy. Unlike the conventional photovoltaic effect, no Dirac delta for the conservation of momentum is present here. Hence, the summation over all initial states in the valence band and over all final states in the conduction band is not restricted by the conservation of momentum (pseudomomentum), which sharply increases the probability of interband transitions. To account for this effect quantitatively, one can consider a spherical Au nanoparticle with a radius *a* embedded in a perovskite layer—cf. Figure 3.

On these metallic nanoparticles, surface plasmons are excited by incident sunlight photons (mostly a dipole mode for nanoparticles with a size much lower than the plasmon resonance wavelength). The absorption of photons by metallic nanoparticles is size-dependent and achieves its maximum at the so-called plasmonic size of metallic nanoparticles [17]. The plasmonic size for Au nanoparticles in a vacuum is ca. 50 nm for the radius of the nanosphere, which is in good agreement with the dipole approximation range, as the plasmonic resonance is typically at ca. 500 nm. With a metallic nanosphere of this size, the radiation (and absorption) of plasmons is extreme, i.e., Lorentz friction dominates the plasmon damping at such dimensions. Lorentz friction is the measure of the radiation of oscillating charges (here electrons) [38,39]. This is because the radiation losses of plasmon oscillations vary nonmonotonically with metallic nanoparticle size. For extremely small metallic grains these losses are negligible but they sharply increase with the cube of the nanoparticle radius and are saturated at the largest value of radiation for plasmonic size (at a radius ca. 50 nm); see [17], Chapter 5. For larger metallic particles this Lorentz friction radiation again diminishes and tends to have a low value in bulk metal. Such a nonmonotonic size-dependence is caused by the Lorentz friction term, which is proportional to the third-order time derivative of the oscillating dipole of the plasmon [38,39] and which means that plasmons are anharmonic oscillations. This behavior is illustrated in Figure 2, in which the damping rate and the resonant frequency of the surface plasmon dipole mode are plotted versus the Au nanoparticle radius. We can see in this figure that the radiation of plasmons becomes extreme for radii near 50 nm for Au nanoparticles.

Due to the symmetry of emission and absorption, for nanoparticles of plasmonic size, we can also observe the extreme absorption of photons by plasmons in the case of these nanoparticles. Therefore, these nanoparticles very efficiently capture incident sunlight photons and quickly radiate the energy. However, if the nanoparticle is surrounded by a semiconductor medium (perovskite in this case), then an additional channel of energy transport from plasmons to electrons is opened. This channel corresponds to the strong coupling of band electrons to plasmons in the near-field zone of plasmon radiation. We demonstrate below (after [17]) that this channel is even more effective than Lorentz friction into free space and in practice all energy quickly flows into the semiconductor without radiation losses.

We refer here to [17] for the relevant formalism and only remind the reader here of the final result regarding the probability of electron inter-band transitions induced by plasmons.

This probability per time unit is as follows,

(1) $$\begin{array}{c} \delta w=\frac{4}{3}\frac{\mu^{2}\left(m_{n}^{*}+m_{p}^{*}\right)2\left(\hbar \omega -E_{g}\right)e^{2}D_{0}^{2}}{\sqrt{m_{n}^{*}m_{p}^{*}}2\pi \hbar^{5}\varepsilon^{2}}\int_{0}^{1}dx\frac{sin^{2}\left(xa\xi \right)}{{\left(xa\xi \right)}^{2}}\sqrt{1-x^{2}}\\ =\frac{4}{3}\frac{\mu^{2}}{\sqrt{m_{n}^{*}m_{p}^{*}}}\frac{e^{2}D_{0}^{2}}{2\pi \hbar^{3}\varepsilon^{2}}\xi^{2}\int_{0}^{1}dx\frac{sin^{2}\left(xa\xi \right)}{{\left(xa\xi \right)}^{2}}\sqrt{1-x^{2}}, \end{array}$$

where $\mu =\frac{m_{n}^{*}m_{p}^{*}}{m_{n}^{*}+m_{p}^{*}}$ expresses the reduced mass. The parameter $\xi =\frac{\sqrt{2\left(\hbar \omega -E_{g}\right)\left(m_{n}^{*}+m_{p}^{*}\right)}}{\hbar }$, introduced above, allows the consideration of two limiting cases for the size of metallic nanoparticles, ξa≪1 (small nanoparticles) and ξa≫1 (large nanoparticles). Equation (1) finally attains the form,

(2) $$\begin{array}{c} \delta w=\left\{\begin{array}{c} \frac{4}{3}\frac{\mu \sqrt{m_{n}^{*}m_{p}^{*}}\left(\hbar \omega -E_{g}\right)e^{2}D_{0}^{2}}{\hbar^{5}\varepsilon^{2}},\;\mathrm{for}\;a\xi \ll 1,\\ \frac{4}{3}\frac{\mu^{3/2}\sqrt{2}\sqrt{\hbar \omega -E_{g}}e^{2}D_{0}^{2}}{a\hbar^{4}\varepsilon^{2}},\;\mathrm{for}\;a\xi \gg 1. \end{array}\right. \end{array}$$

Through the direct calculation of the parameter ξ for perovskites, we note that for these materials and metallic nanoparticles with sizes larger than 1 nm for the radius *a*, the lower limit in the above equation applies.

In the conventional photo-effect, when the electron inter-band transitions are induced by a plane wave of incident photons, the similar probability of interband transitions is as follows [42],

(3) $$\begin{array}{c} \delta w_{0}=\frac{4\sqrt{2}}{3}\frac{\mu^{5/2}e^{2}}{m_{p}^{*2}\omega \varepsilon \hbar^{3}}\left(\frac{\varepsilon E_{0}^{2}V}{8\pi \hbar \omega }\right){\left(\hbar \omega -E_{g}\right)}^{3/2}. \end{array}$$

One can estimate the number of incident photons with the energy ℏω corresponding to the e-m wave sunlight with the frequency ω in the volume *V*, $\left(\frac{\varepsilon E_{0}^{2}V}{8\pi \hbar \omega }\right)$, where $E_{0}$ is the amplitude of the electric field of this component of the sunlight spectrum. Hence, the probability of the immediate absorption of a single photon by the perovskite per time unit equals [42],

(4) $$q_{0}=\delta w_{0}{\left(\frac{\varepsilon E_{0}^{2}V}{8\pi \hbar \omega }\right)}^{-1}=\frac{4\sqrt{2}}{3}\frac{\mu^{5/2}e^{2}}{m_{p}^{*2}\omega \varepsilon \hbar^{3}}{\left(\hbar \omega -E_{g}\right)}^{3/2}.$$

The probability of sunlight energy absorption in perovskite, mediated by plasmons and normalized per single incident photon, is equal to the the product of δw as in Equation (2) and the number of metallic nanoparticles *N*, finally divided by the photon density,

(5) $$q_{m}=\beta N\delta w{\left(\frac{\varepsilon E_{0}^{2}V}{8\pi \hbar \omega }\right)}^{-1},$$

with an additional factor β for all effects not directly included in the above derivation.

The ratio $\frac{q_{m}}{q_{0}}$ gives us the spectral information on the advantages in terms of photon capture if it is mediated by plasmons in metallic nanoparticles with a radius of *a*. In Equation (2) $D_{0}$ indicates the amplitude of dipole surface plasmons oscillating on the metallic nanoparticles. This amplitude is linked to the amplitude $E_{0}$ of the incident sunlight component with frequency ω. This linkage is elucidated in the next section.

The above theoretical formalism for plasmon-mediated absorption of photons was applied in the present study to examine this effect in metallized perovskite solar cells with specially tailored metallic components in order to activate and strengthen this plasmonic channel in perovskite cells. This is important in view of the very poor plasmonic-mediated absorption in perovskite cells metallized by spherical golden or silver nanoparticles [28].

### Steady Transfer of Sunlight Energy to Perovskite Electrons via Plasmons in Metallic Nanoparticles

Oscillations of plasmons in metallic nanoparticles induced by incident sunlight can be described in terms of forced and damped oscillators [17]. In the steady-state conditions of such an oscillator, all the energy incoming to plasmons from sunlight photons is transferred to the surrounding semiconductor (perovskite) and to the surrounding space via the e-m wave due to Lorentz friction (and some small fraction is irreversibly transformed into a Joule of heat due to Ohmic losses of oscillating electrons in metallic components). As a result, the amplitude of plasmon oscillations $D_{0}$ is kept constant in time. For such a steady regime of a damped and forced oscillator, its amplitude $D_{0}\left(\omega \right)$ is governed by the conventional spectral factor,

(6) $$f\left(\omega \right)=\frac{1}{\sqrt{{\left(\omega_{1}^{2}-\omega^{2}\right)}^{2}+4\omega^{2}/\tau^{2}}},$$

where τ is the damping rate of plasmon and $\omega_{1}$ is its resonance frequency. The damping of plasmons causes a red-shift of the resonance (as visible from the maximum of Equation (6)) and reduces the resonant amplitude.

Damping of plasmons, expressed by τ, must include all losses of oscillation energy caused by dissipative Ohmic losses, Lorentz friction losses and the energy transfer to band electrons in perovskite. One can verify that the latter channel dominates the others. This follows from the comparison of damping time rates for the three channels listed above [17].

In order to assess quantitatively the efficiency of the energy transfer from plasmons to band electrons in the surrounding semiconductor we assume an idealized model situation in which the inflow of energy to the substrate semiconductor is equal to the outflow of energy from plasmons oscillating in metallic nanoparticles. In such a model case, the amplitude of the plasmon dipole decreases over time as $D_{0}\left(t\right)=D_{0}e^{-t/\tau^{\prime }}$ and the energy transferred to the semiconductor equals (with δw given by Equation (2) for limit aξ≫1),

(7) $$A=\beta \int_{0}^{\infty }\delta w\hbar \omega dt=\beta \hbar \omega \delta w\tau^{\prime }/2=\begin{array}{c} \frac{2}{3}\frac{\beta \omega \tau^{\prime }\mu^{3/2}\sqrt{2}\sqrt{\hbar \omega -E_{g}}e^{2}D_{0}^{2}}{a\hbar^{3}\varepsilon^{2}}, \end{array}$$

$\tau^{\prime }$ indicates the damping time rate due to coupling of plasmons to electrons, and the parameter β<1 accounts for all effects which were neglected here but which could reduce the transition probability (such corrections could arise, e.g., by virtue of irradiation losses in medium- and far-field zones, not included here). Through the comparison of A with the initial energy of the plasmon oscillations, one can determine the damping rate, $\frac{1}{\tau^{\prime }}$. The initial energy of plasmons equals [17]$B=\frac{D_{0}^{2}}{2\varepsilon a^{3}}$; thus, from A=B we obtain,

(8) $$\frac{1}{\tau^{\prime }}=\begin{array}{c} \frac{4\beta \omega \mu^{3/2}\sqrt{2}\sqrt{\hbar \omega -E_{g}}e^{2}a^{2}}{3\hbar^{3}\varepsilon }, \end{array}$$

and $\tau^{\prime }$ becomes shorter that the time rate for Lorentz friction and for Ohmic electron losses (see [17], Chapter 6).

Therefore, utilizing Equations (6)and (8), the final form for the probability of interband transitions in perovskite mediated by plasmons (i.e., Equations (2) and (5)) is as follows:

(9) $$q_{m}=\begin{array}{c} \beta C_{0}\frac{128}{9}\sqrt{2}\pi^{2}a^{2}\frac{\mu^{3/2}}{m^{2}}\sqrt{\hbar \omega -E_{g}}\frac{e^{6}n_{e}^{2}\omega }{\hbar^{3}\varepsilon^{3}}f^{2}\left(\omega \right), \end{array}$$

Using the above Formula (9) compared with (4) one can illustrate the benefit in terms of the photo-effect caused by plasmons in various materials in dependence on sunlight frequency and on metallic nanoparticle size (of an arbitrary metallic material). The ratio of the photon absorption rate in the semiconductor with and without metallic components, $\frac{q_{0}+q_{m}}{q_{0}}\left(\omega ,a\right)=1+\frac{q_{m}}{q_{0}}\left(\omega ,a\right)$, is explored in the following section for several selected materials.

## 5. Various Routes for the Utilization of the Plasmonic Effect in Perovskite Solar Cells

Various methods have been demonstrated to increase the efficiency of solar cells via metallization. The simplest one is to attach a thin metallic layer on the back of a solar cell which would act as a mirror reflecting photons, transmitting them across the active layer again to the inside of the semiconductor to increase the path of photons penetrating the volume of the cell. Such an increase of the photon path results in an enhancement of the excitation rate of electron–hole pairs inside the semiconductor layer, which ultimately provides a gain in the photocurrent. This simple method can be applied to any type solar cell, in particular to perovskite cells which are manufactured in the thin-layer configuration (the optimal thickness of the perovskite layer is typically ca. 300 nm, thus doubling the photo channelis of particular significance here [40,41]). Moreover, the metallic substrate can play an insulating role, contributing to the cell encapsulation, protecting against environmental degradation.

Another possibility is to add an upper or bottom conducting layer (an upper layer must be sufficiently thin and transparent), which would create a planar waveguide for incident solar photons, together with metallic nanoparticles incorporated into the perovskite layer on the opposite side of the cell. The e-m wave of incident sunlight photons in such a waveguide may hybridize with plasmons in both metallic (conducting) layers, creating a plasmon-polariton wave-type excitation and eventually also enhancing the interactions of initial photons with perovskite band electrons. Such a waveguide arrangement in the case of a conventional semiconductor cell (Si) revealed a large increase in the cell’s efficiency [43].

Finally, mediation by dipole modes of surface plasmons in metallic nanoparticles may increase the photon absorption due to a quantum effect. This channel of the plasmonic PV effect has been investigated during the last few years [18,19,20,21,22,23,24,25,26] in various cells. It has been experimentally demonstrated that metallic nanoparticles (mostly consisting of the noble metals Au and Ag, and also Cu) deposited on the surface of the semiconductor (in p-n junction cells, such as Si, CIGS or GaAS cells) cause a large increase in the efficiency of the photo-effect—for a review of this effect in p-n junction solar cells and of the related theoretical model, see [17], Chapters 6–8. Based on the experiments presented there, it follows that even relatively low surface concentrations of metallic components (ca. $10^{8}$ nanoparticles per square centimeter [18]) greatly increases the efficiency of metallized cells. The focusing of the electric field of the sunlight e-m wave near the curvature of a metallic particle, which can be classically calculated using, e.g., the Comsol system, contributes only a small fraction (ca. 5%) to the increase in photon absorption. The dominant part (ca. 95%) of the observed efficiency increase due to plasmons is quantum in nature and can be accounted for according to the Fermi golden rule scheme (as mentioned in Section 4) [17]. Thus, the classical treatment of the plasmonic photovoltaic effect, in which only the local concentration of the electric field of the incident light wave is considered in the vicinity of the metallic component’s curvature, is highly insufficient. Such a classical approach is conventionally used in the modeling of plasmonic effects via the solution of the Maxwell–Fresnel classical electrodynamic problem, frequently by means of the numerical finite element method for the solution of differential equations based upon, e.g., the Comsol system. Thus, the results of Comsol or similar simulations should be treated with caution as they may be highly misleading unless they are corrected for the quantum effects described above.

The application of metallic nanoparticles to enhance the efficiency of conventional solar cells with p-n junctions, such as Si or CIGS cells, has been demonstrated experimentally and explored theoretically. There is a problem, however, with the durability of metallic nanoparticle coverage. The application of a special protecting layer is not easy and increases costs; thus, the metallization of conventional p-n junction cells is not widely applied at present. Metallic nanoparticles must be deposited on the top surface of the semiconductor. This is in contrast to perovskite cells, for which low-temperature chemical crystallization from liquid precursors, even through ink-jet printing, allows for the submergence of metallic components inside the semiconductor layer without the need to fix them using additional protecting layers. This makes the metallization of perovskite cells more promising than that of conventional p-n junction cells. Note also that, despite using noble metals for metallic components, the cost of materials is in fact very low, because of the low concentration of nanoparticles required (the cost is not more than 1 USD per 1 m^2^ of a cell).

**Figure 4:**
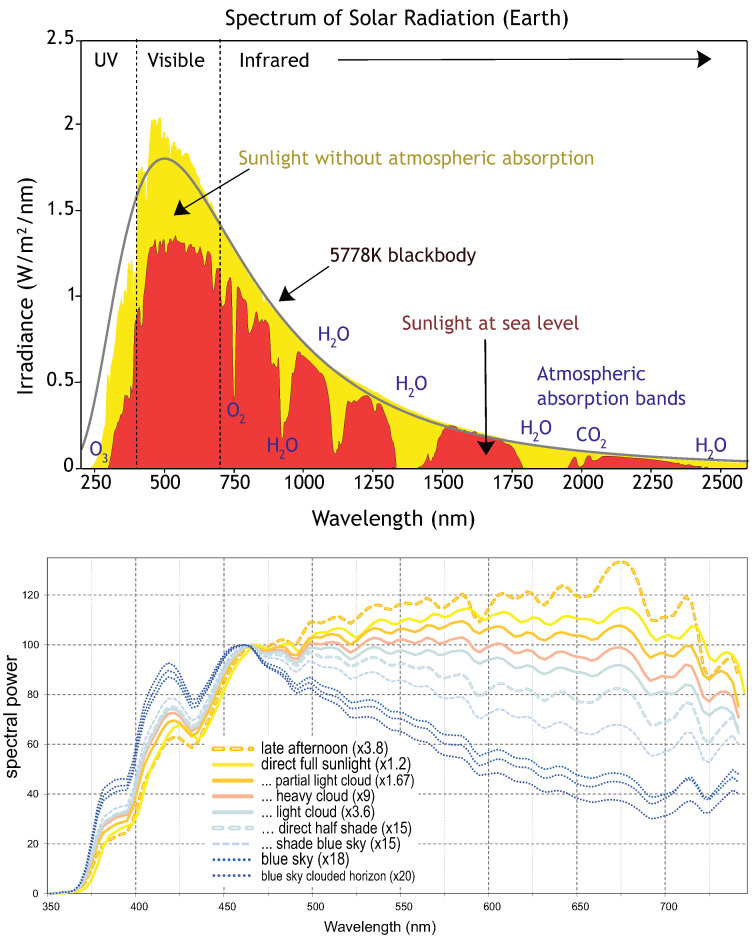
Sunlight spectrum at sea level versus the full radiation above the atmosphere (**upper** panel). Proportion of dispersion for additional damping of sunlight by atmospheric illumination conditions (**lower** panel).

The most important parameter for solar cells is the forbidden gap in the used semiconductor. The Shockley–Queisser efficiency limit [30] shows that the maximum of the efficiency is attainable at the bandgap equal to ca. 1.34 eV, at which the utilization of the full sunlight spectrum is optimal. This spectrum mostly covers the visible light and infrared fraction, whereas the UV component is reduced by the atmosphere; cf., Figure 4, upper panel. Additionally typical atmospheric conditions such as clouds reduce the solar intensity mainly in the red and infrared regions of the spectrum (Figure 4, lower panel).

Perovskites have the structure of ABX${}_{3}$, where A${}^{+}$ is an organic cation (a typical example is CH${}_{3}$NH${}_{3}^{+}$), B${}^{2+}$ is a metallic cation (Pb, Sn, Ge) and X${}^{-}$ is a halogen anion. The most popular perovskite utilized in photovoltaics is methylammonium lead triiodide, CH${}_{3}$NH${}_{3}$PbI${}_{3}$, which has high mobility of charges (the effective diffusion length for both electrons and holes exceeds 100 nm). The mixed methylammonium lead halide CH${}_{3}$NH${}_{3}$PbI${}_{3-x}$Cl${}_{x}$ may have an even longer mean free path for carriers (of the order of μm), so variation of the halogen anion would not worsen the electrical quality of the perovskite. Moreover, the bandgap in perovskites can be tailored according to the chemical composition and varies between 1.2 and 2.3 eV. Lead, which is hazardous for the environment, can be substituted by changing the chemical composition, but this worsens the cells’ photovoltaic properties. The replacement of lead with lowly-toxic elements could include the use of tin/germanium-halide perovskites and bismuth/antimony-halides with perovskite-like structures [11,12,13].

The family of perovskite materials displays variations in the forbidden gap with changes in chemical composition, as illustrated in Figure 1 and in Table 1. The possibility of designing thin-film hybrid chemical perovskite solar cells with large or small gaps in the perovskite absorber can be utilized to construct tandem perovskite-perovskite cells which are able to surpass the Shockley–Queisser efficiency limit through a much better accommodation of the sunlight spectrum in both its shorter and longer wavelength regions. Wide bandgap perovskites are used as the top sub-cells in tandem perovskite-perovskite devices, whereas the bottom layer is composed of a smaller-gap perovskite. In methylammonium lead halide perovskites (MAPbX${}_{3}$, CH${}_{3}$NH${}_{3}$PbX${}_{3}$, where X = I, Br or Cl), the bandgap of MAPbI${}_{3–x}$Br${}_{x}$ can be tuned from about 1.6 eV to 2.3 eV (cf., Figure 1). However, the performance of the devices also changes greatly in accordance with the Br-I mixing ratio. The open-circuit voltage (VOC) and fill factor (FF) increases for moderate Br ratios (*x* < 0.2), but the VOC drops rapidly at Br ratios higher than 0.2. Nevertheless, the application of Br strongly improves the stability of perovskites, especially against humidity.

A way to reduce the bandgap of the perovskite is through the substitution (even partial substitution) of Pb${}^{2+}$ with Sn${}^{2+}$ (cf., Figure 1, left panel). However, a major obstacle in the development of Sn-based perovskites is their inconveniently high carrier density and short carrier lifetime, caused by the rapid oxidation of Sn${}^{2+}$ to Sn${}^{4+}$. The development of low-bandgap perovskites with Sn-Pb alloy was reported in [44].

Large-gap perovskites utilize the UV part of the solar spectrum, i.e., for $E_{g}=2.3$ eV photons must have a wavelength shorter than 269 nm (UV region), whereas for a lower-gap perovskite with $E_{g}=1.6$ eV the limiting photon wavelength is 387 nm, i.e., it overlaps with the violet part of spectrum (the visible light sector is 380–750 nm). Both these cases are much worse in comparison to Si cells, with $E_{g}=1.14$ eV and a limiting wavelength of 543 nm. The small overlap of the active region of large- and medium-gap perovskites with the solar-light spectrum reduces the efficiency of cells with these materials.

In order to strengthen the interband excitations in these materials by means of plasmons, one must accommodate plasmonic resonance into the active region in the perovskites. The maximal value of the plasmon resonance in metallic nanoparticles can be widened via various factors, including the damping of plasmons (resulting in the widened Lorentzian dispersion) and size and shape inhomogeneity (the dependence of the resonant frequency for Au versus the nanosphere radius is plotted in Figure 2, in which the red-shift with growing nanoparticle size is visible). The dielectric coating also influences plasmonic resonance in nanoparticles and causes a red-shift in their resonance [28].

**Figure 5:**
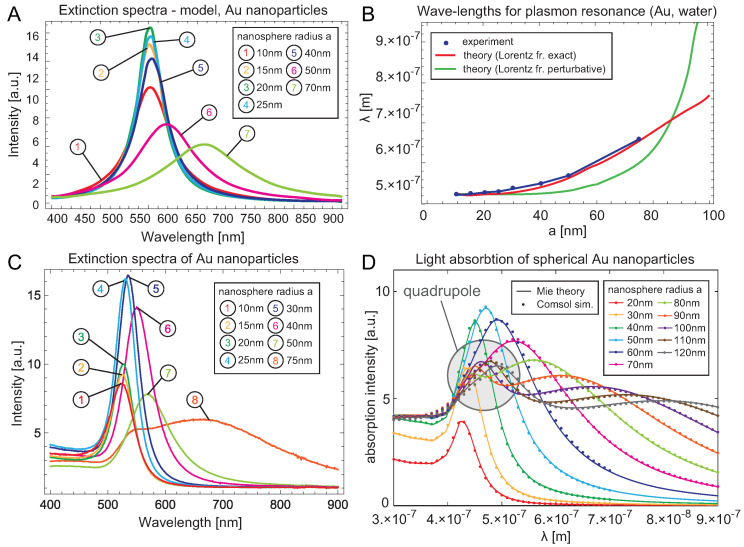
Spectral dependence of light absorption in a spherical Au nanoparticle with a varying radius. (**A**). Theoretically-found dependence in the model of the damping of plasmons in Au nanospheres on size-dependent Lorentz friction [17]. (**B**). Comparison of the Lorentz friction model with experimental results. (**C**). The experimental data for the absorption spectra of Au nanoparticles with varying radii reveal very good agreement with the Lorentz friction model (**A**) [17]. The most pronounced dipole mode of surface plasmons is dominant at sufficiently low sizes of the particle, when dipole approximation holds (i.e., when the resonant wave-length is much larger than the radius of the nanoparticle). For larger nanoparticles, the quadrupole surface plasmon resonance, slightly blue-shifted, additionally appears (indicated by the shadowed region in panel (**D**), where the corresponding Comsol simulation is presented [45]). The arising of the quadrupole mode is also visible in the experimental data in (**C**) for an Au nanosphere with a radius equal to 75 nm.

The surface plasmon resonant frequency depends on the dielectric permittivity ε of the surroundings, according to the formula $\omega_{1}\left(l\right)=\omega_{p}\sqrt{\frac{l}{\varepsilon (2l+1)}}$ (found upon random-phase approximation [17] for each mode of surface plasmons, distinguished in spherical symmetry with multipole *l*—integer number; for l=1 the dipole mode gives the so-called Mie frequency), and $\omega_{p}=\sqrt{\frac{n_{e}e^{2}}{\varepsilon_{0}m}}$ is the plasmon frequency in bulk metal (cf. Table 2), $n_{e}$ is the density of electrons in a metal, *e* is the electron charge, *m* is the electron mass and $\varepsilon_{0}$ is the dielectric constant. The integer *l* enumerates multipole modes for a metallic nanoparticle with a spherical geometry (l=1 gives the dipole mode, l=2 the quadrupole one, and so on). The possibility of exciting multipole higher modes increases with the nanoparticle radius *a*. For a∈(5,60) nm, only the dipole mode can be excited by the e-m wave in Au because of the dipole approximation constraint, and a quadrupole mode (slightly blue-shifted) arises in particles with radius greater than 60 nm [17]. If damping of plasmons due to Lorentz friction is neglected, then the quadrupole mode occurs earlier in approximate numerical simulations—at a radius ∼40 nm. See Figure 5, in which a Comsol simulation without Lorentz friction is shown [45]. However, the blue-shift of this mode is small and the contribution of the quadrupole mode is much lower in comparison to the dipole one.

**Figure 6:**
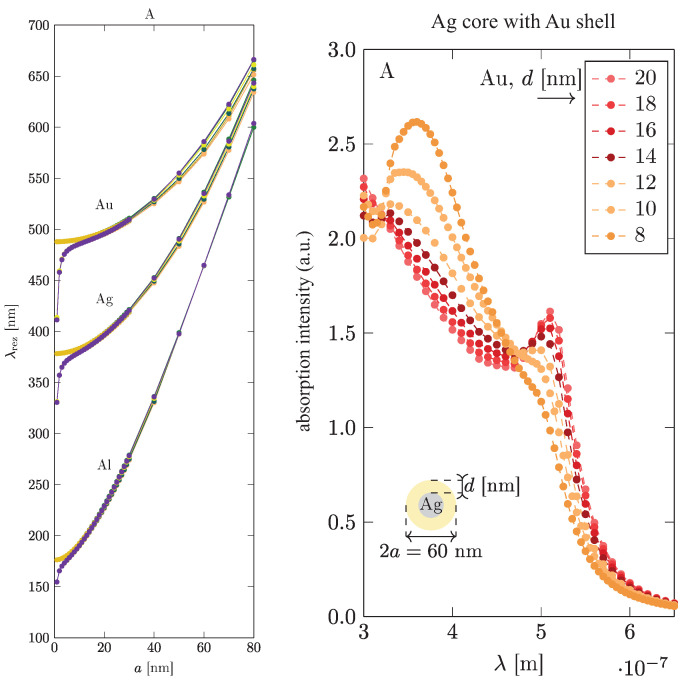
(**left**) Dependence of the surface plasmon resonance on material. A strong reduction in the resonance wavelength can be achieved through the application of Fe or Al nanoparticles (small splitting in resonance curves is related to various approximations of plasmon damping used in the simulation). (**right**) By adjusting the shell-core bi-metal nanoparticle composition, it is possible to adjust precisely the plasmon resonance in metallic components to its optimal value for the plasmon photovoltaic effect in perovskite cells with varied gorbidden gaps [45] (Comsol simulation).

**Figure 7:**
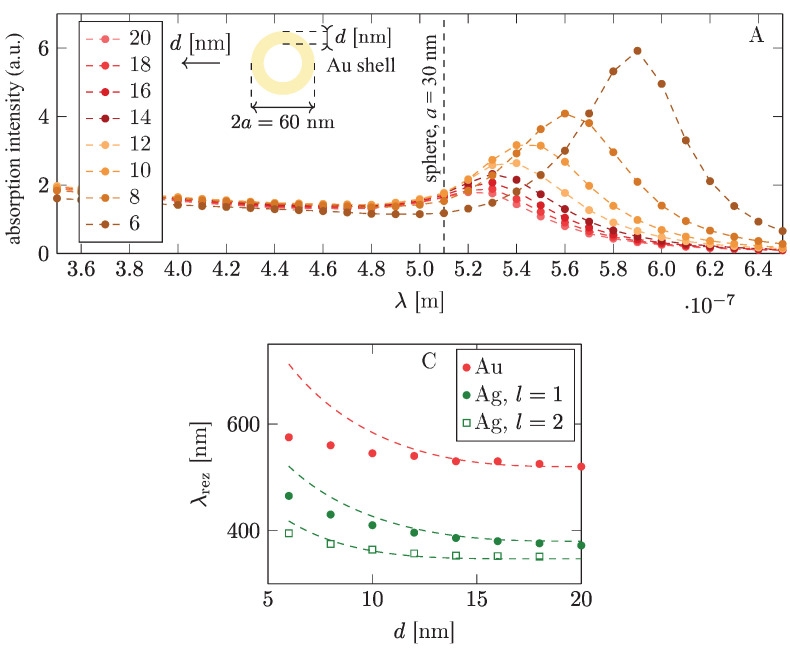
The dipole surface plasmon resonance in a single shell-type nanoparticle versus its geometry. In lower panel, l=1 and l=2 for Ag denote the dipole and quadrupole modes, respectively [45] (Comsol simulation).

**Figure 8:**
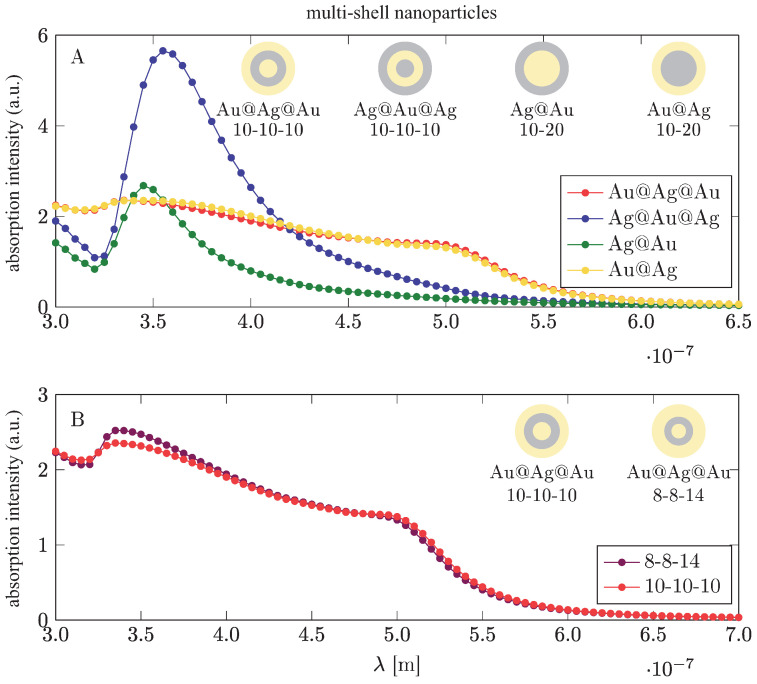
Flattening and elongation of the dipole surface plasmon resonance in multi-shell-core metallic nanoparticles. Thicknesses of shells are shown in nanometers [45] (Comsol simulation).

In order to shift the plasmon resonance more strongly in metallic components, the variation of the metallic material is more effective (cf., Figure 6, left panel, and Table 2), as is the application of multi-shell particles using different materials for particular shells (cf., Figure 6, right panel, Figure 7 and Figure 8; the simulations were performed in Comsol).

Based on the above, it follows that an appropriate tailoring of the material, size and multi-shell structure of metallic nanoparticles allows for the better accommodation between plasmonic resonance, the solar spectrum and the semiconductor bandgap. For perovskites with a relatively large gap, the metallic components should have a blue-shifted plasmonic resonance. This leads to a preference for smaller-sized Au, Ag or Cu nanoparticles, Ag@Au or Au@Ag@Au, Au@Al or Au@Fe core-shell metallic nano-components. The application of dielectric coatings is also recommended to isolate metal from the material of the electron or hole transport layer and of the perovskite absorber to preserve the layer morphology and protect against the capturing of diffusive electrons or holes, as well as to deactivate recombination centers for excitons. However, rather thin dielectric coatings are recommended here in order to avoid inconvenient red-shifts due to thick dielectric layer.

**Figure 9:**
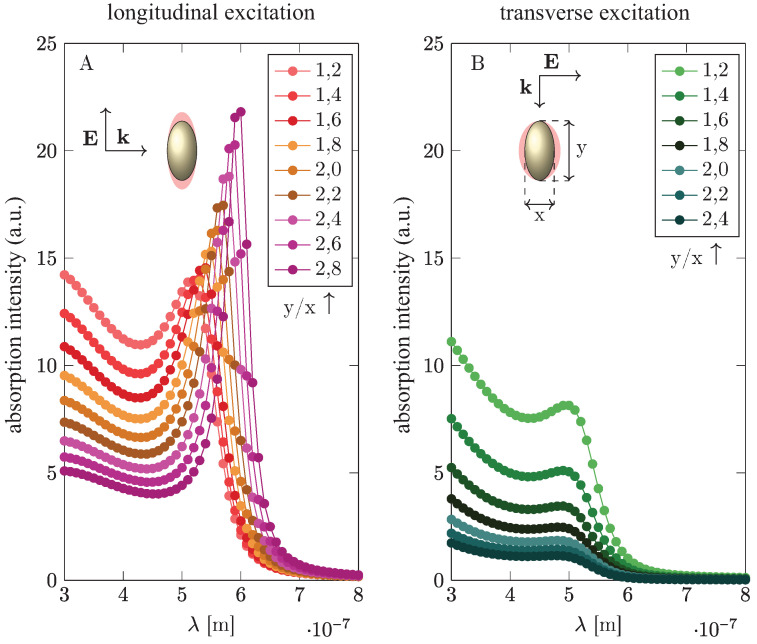
Plasmon double resonance in elongated metallic nanoparticles. (**left**) The resonance for dipole oscillations is depicted along the longer axis. (**right**) The resonance for oscillations is depicted along the shorter axis—the frequency difference between both polarizations increases as the aspect ratio increases [45] (Comsol simulation).

Another metallization strategy is to apply elongated nanoparticles with a plasmon resonance that is split into two parts, corresponding to plasmon oscillations along longitudinal and transverse directions with respect to the longer axis of the nanoparticle.The oscillations along the shorter axis are of higher energy in comparison to the longer one and the difference grows with the increase in the aspect ratio; cf., Figure 9. In this figure, presenting the results of a Comsol simulation, we can see that the elongated geometry conveniently allows one to match the blue and red parts of the sunlight spectrum. For perovskites with larger gaps, such an opportunity to enter the blue and UV region is of significance, and indeed it has been demonstrated experimentally that the a record plasmonic increase in perovskite cell efficiency (40% of the relative growth) was achieved through the application of elongated Au particles [16]. It has been also demonstrated experimentally that the application of Ag nanoparticles with small dimension and a large blue-shift of plasmonic resonance strongly enhances light absorption in CH${}_{3}$NH${}_{3}$PbI${}_{3}$ perovskite cells [46]. The application of Au nanorods with double plasmon resonance has been also demonstrated in perovskite cells in synergy with MgO, resulting in a ca. 20% increase in relative efficiency [47].

Taking into account that in perovskite solar cells the electrical intrinsic plasmonic effect is dominant, which is connected with the reduction of the binding energy of excitons created via the mediation of plasmons [14,28,29], one can expect that double plasmon resonances in elongated and/or multishell nanoparticles would be especially convenient. The electrical intrinsic plasmonic effect is caused by the nonzero relative momentum of electron–hole pairs excited by plasmons in the perovskite. The shift in the binding energy of excitons due to plasmons can be estimated according to the formula [28,29]

(10) $$\Delta E=\frac{\int d^{3}k_{1}\int d^{3}k_{2}\frac{\hbar^{2}q^{2}}{2\mu }w\left(k_{1},k_{2}\right)}{\int d^{3}k_{1}\int d^{3}k_{2}w\left(k_{1},k_{2}\right)}=\frac{\frac{\hbar^{2}}{2\mu }\int_{0}^{1}dx\frac{sin^{2}\left(xa\xi \right)}{a^{2}}\sqrt{1-x^{2}}}{\int_{0}^{1}dx\frac{sin^{2}\left(xa\xi \right)}{{\left(xa\xi \right)}^{2}}\sqrt{1-x^{2}}},$$

where $q=k_{1}-k_{2}$ indicates the difference in the pseudomomenta of the initial and final band states of an electron excited from the valence to the conduction band of the perovskite by plasmons from metallic nanoparticles (where $w(k_{1},k_{2}$) indicates rhe probability per time unit of the interband transition of an electron between $k_{1}$ and $k_{2}$ in the valence and conduction bands, respectively, $\xi =\frac{\sqrt{2\left(\hbar \omega -E_{g}\right)\left(m_{n}^{*}+m_{p}^{*}\right)}}{\hbar }$). Equation (10) allows for a theoretical quantitative assessment of the intrinsic electric photovoltaic plasmonic effect [14] and admits for the optimization of this effect in a model through the variation of plasmonic parameters.

It is worth emphasizing that through the application of prolate-shaped metallic nanoparticles it is possible to optimize simultaneously the absorption of sunlight energy via plasmons (the optical plasmon photovoltaic effect) and the intrinsic electric plasmonic effect, which will together benefit the total increase in the efficiency of metalized perovskite solar cells, though separately requiring the usage of nanoparticles with different plasmonic parameters. Absorption enhances the voltage in the cell, whereas the electrical effect causes the photocurrent to increase [28]. The optimal efficiency increase of a perovskite cell will take advantage of both these channels by appropriately accommodating both plasmon resonances in prolate-shaped nanoparticles.

**Figure 10:**
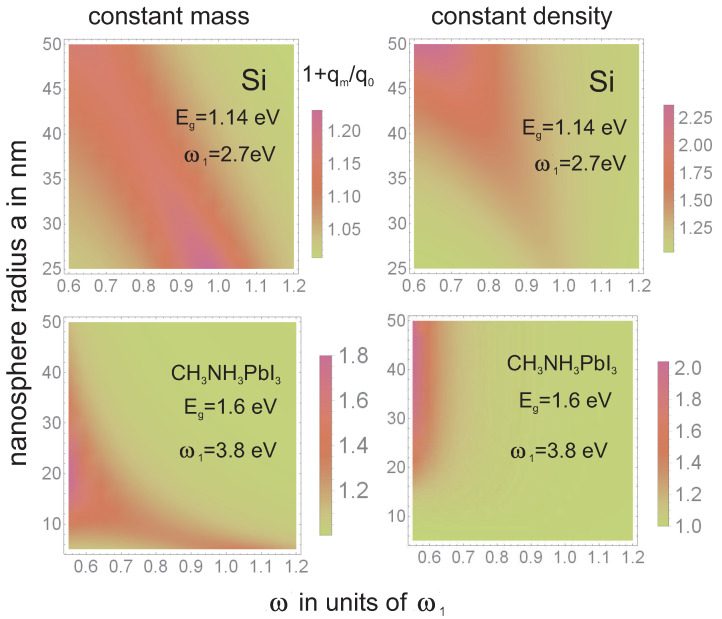
Density plots of $1+\frac{q_{m}}{q_{0}}$ as the function of frequency $\omega /\omega_{1}$ of incident photons and of nanoparticle radius *a*, according to Equations (4) and (9) (this function displays an increase in the light absorption aided by plasmons and normalized to direct photo-absorption without metallic component mediation), for comparison, of an Si cell (with plasmonic Au nanoparticles with dipole frequency 2.7 eV) and of a perovskite cell (with an adjusted plasmonic frequency 3.8 eV to the gap 1.6 eV). Two situations are displayed—with a constant total mass of metals at varying sizes of nanoparticles (**left**) and with constant metallic nanoparticle concentrations (**right**) (Mathematica software).

**Figure 11:**
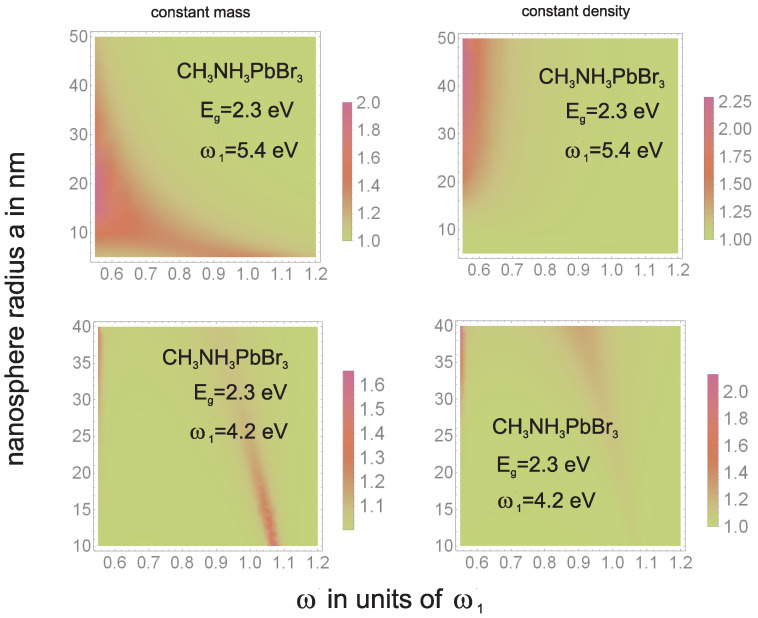
Density plots of $1+\frac{q_{m}}{q_{0}}$ as a function of frequency $\omega /\omega_{1}$ of incident photons and of nanoparticle radius, *a*, in accordance with Equations (4) and (9) (this ratio displays an increase in the light absorption, aided by plasmons, normalized to the photo-absorption without the mediation of metallic components) for a large-gap perovskite cell (adjusted to a large-gap plasmonic frequency of 4.2 eV and 5.4 eV for the gap of 2.3 eV). The graphs indicate that it is possible to increase the absorption of perovskite cells in the UV region using metallic nanoparticles with smaller radii than of those needed for Si cells; see Figure 10. For higher-gap perovskites, a higher energy plasmon resonance than that obtained in Au nanoparticles is needed. The latter is caused by the frequency cut-off of the gap, whereas a smaller radius is required to compensate for the growth of the effective masses of electrons and holes in perovskite compared to Si cells to maintain a similar damping rate to that given in Equation (8). Constant density refers to a fixed surface density of metallic nanoparticles, at $5\times 10^{9}$/cm${}^{2}$ regardless of the variation in nanoparticle size, whereas constant mass indicates a fixed total mass of metal, distributed in the form of nanoparticles with varying sizes and thus with larger concentrations for smaller radii (Mathematica software).

**Figure 12:**
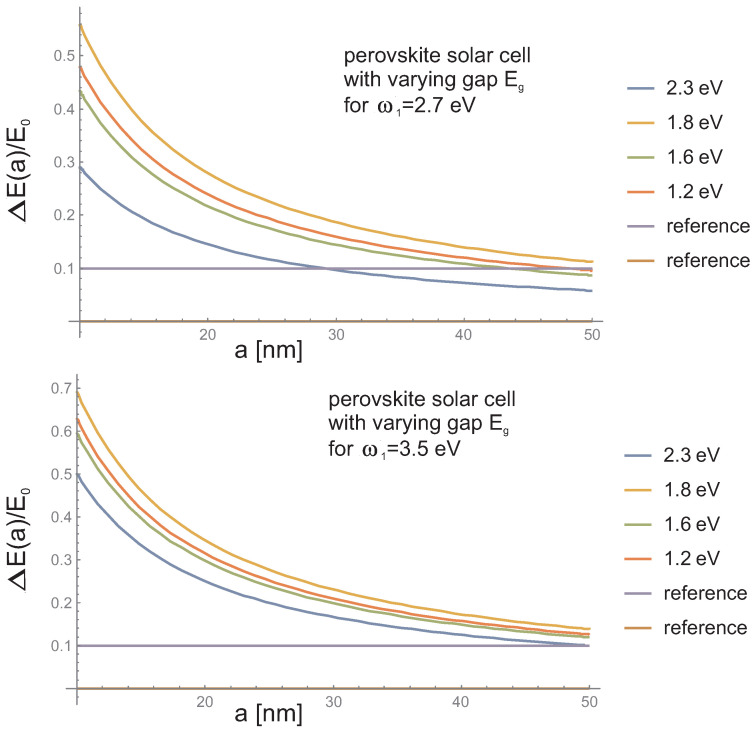
Reduction in the binding energy of excitons created by plasmons versus the size of metallic nanoparticles for several representatives of the perovskite family with various forbidden gaps ($E_{g}=2.3,\;1.8,\;1.6,1.2$ eV) and for metallization with Au nanoparticles ($\omega_{1}=2.7$ eV)—upper panel, and for blue-shifted plasmon resonance ($\omega_{1}=3.5$ eV)—lower panel. The reduction in binding energy $\Delta E\left(a\right)/E_{0}$ is normalized according to the energy of electrons with a reduced mass $\mu =\frac{m_{n}^{*}m_{p}^{*}}{m_{n}^{*}+m_{p}^{*}}$ at the edge of the Brillouin zone, $E_{0}=\frac{p^{2}}{2\mu }$, for $p=\frac{\hbar \pi }{l}$ and l=5 nm—the averaged linear size of the elementary cell of perovskite. The reduction in exciton binding energy was calculated according to Equation (10) (Mathematica software).

To illustrate how the tailoring of plasmon resonance may influence the overall absorption of photons, we show in Figure 10 and Figure 11 the theoretically estimated absorption growth $1+\frac{q_{m}}{q_{0}}\left(\omega ,a\right)$ according to Equations (4) and (9). In Figure 12 we show how the intrinsic electrical plasmonic effect depends on the variation of the forbidden gap in the perovskite used and on the metallization type. We can see that perovskites with a smaller forbidden gap are more convenient, and the disadvantage caused by the large gap can be efficiently reduced through the application of metallic components with blue-shifted plasmon resonance.

In order to avoid the worsening of carrier mobility and to reduce the parasitizing increase in the local recombination of excitons on the bare metal surface of nanoparticles, an application in perovskites of core-shell nanoparticles with an outer coating by means of an appropriately designed insulating dielectric layer (e.g., of SiO${}_{2}$) is proposed. Such a dielectric spacer separates surface plasmons in a metallic core from charge carriers (electrons, holes and excitons) in the surrounding semiconductor medium and, on the other hand, allows for the tuning of plasmon resonance by varying the insulating layer thickness and its dielectric permittivity. In perovskite cells, the application of core-shell metallic components coated with protective dielectric layers significantly improved efficiency [14,15,16], but evidence has shown that the intrinsic electrical channel of the plasmon PV effect plays the central role here, in contrast to metallized conventional p-n junction cells, in which the optical absorption PV channel was dominant [17,28]. The minor role of the optical plasmonic absorption channel in metallized perovskite cells is related to the large forbidden gaps in the perovskites used and the large effective masses of the carriers (both of electrons and holes) cf. Table 3, which together lead to the incompatibility of perovskite electric susceptibility to dipoles with a resonance energy that is too low (in spherical nanoparticles of Au). Thus, a better tailoring of metallic nano-components by altering their multi-shell structure and using an elongated shape may appropriately shift or split the plasmon resonance, enhancing the overlapping of plasmon resonances with perovskite susceptibility. In this way it is possible to enhance the absorption PV channel as well and to exploit fully the potential of the plasmonic photovoltaic effect.

Overly dense metallic additives can act, however, as defects in the crystal structure of the perovskite, reducing the mean free paths of excitons and of charged carriers, liberated from excitons at the interface with the charge transport layer. To minimize such effects, a coating of metallic nanoparticles with an outer dielectric layer is applied, reducing their surface activity, but they still contribute negatively as structure defects. Researchers have also identified another reason for reductions in efficiency—structural imperfections [48] related to hydrogen vacancies, which frequently occur in CH${}_{3}$NH${}_{3}^{+}$ components in the perovskite. To remedy such defects, the authors in [48] proposed substituting the methylammonium component with the formamidinium group CH(NH${}_{2}$)${}_{2}^{+}$, which exhibits an electron-capture coefficient at hydrogen vacancies that is three orders of magnitude lower.Formamidinium cations or halides may partially or fully conveniently replace methylammonium halides in the formation of perovskite absorber layers in photovoltaic cells.

To improve operation and efficiency, new solutions for the electron transport layer have also been proposed. SnO${}_{2}$ doped with crystalline polymeric carbon nitrides (cPCN) allowed for high electron mobility in this layer (more than $3\times 10^{-3}$ cm${}^{2}$/(Vs)). The perovskite solar cell based on SnO${}_{2}$-cPCN showed negligible J–V hysteresis and efficiency beyond 23% [49].

In Figure 10 and Figure 11 one can note that, to increase the absorption of photons in perovskite cells via the plasmonic effect, the application of either low-gap perovskite or metallic particles with higher resonant frequency is useful.The latter (blue-shifted resonance) can be achieved through various strategies including multishell bi-metallic particles (cf., Figure 6 and Figure 8) or by elongating their shape (cf., Figure 9). In the experiment reported in [16] the record relative increase in efficiency (of 40%) due to the application of gold rods with strongly blue-shifted transverse plasmon resonance confirms such an opportunity (the activation of the absorption plasmonic channel was confirmed by the increase in the voltage in the U-I that was characteristic of the cell in that experiment). In the application of spherical particles, the absorption component of the plasmonic effect is quenched, as is visible in the experiments reported in [14,15] (this is also evident in the dominant increase in the photocurrent only in U-I characteristics related with the electrical channel of the PV plasmon effect). Although the electric effect also provides a relatively large increase in efficiency (preferably with a medium blue-shift of the plasmon resonance; cf., Figure 12), the two-times-larger efficiency increase observed in [16] in comparison to the experiments reported in [14,15] clearly reveals the potential of the simultaneous activation of both PV plasmonic channels in perovskite cells through the tailoring of the shape and material composition of metallic admixtures.

Finally, it is also worth mentioning a prospective application of perovskite cells in space and on the Moon. In circumstances without oxygen and water, ultra-thin perovskite cell stripes can be more durable without encapsulation. They could be printed in place using liquid precursors that are easy to transport using rockets. Moreover, kilometer-long stripes on the Moon could be easily exploited and recycled without any atmospheric perturbation. Additionally, in space or on the Moon, the different sunlight spectrum, with a larger component of UV light, is conveniently for larger-gap semiconductors and for photo-active agents, which additionally supports the usability of perovskite cells that have been suitably metallized.

## 6. Summary

In this study, we explored different pathways towards increasing the efficiency of perovskite photovoltaic cells via nano-scale metallization. Since perovskite cells function as a hybrid chemical cell in a different way than conventional p-n junction cells, the plasmonic photovoltaic effect is different in each type of cell. In p-n junction cells (e.g., Si-based cells) it is of an optical type, i.e., plasmonic components mediate in the capture of incident sunlight photons. In perovskite cells, however, an intrinsic electrical plasmonic effect related to the lowering of the binding energy of excitons is dominant. Because of the large gap and heavy masses of carriers, the optical plasmonic effect is weak and inconveniently shifted to the UV spectral region in perovskite absorbers. Nevertheless, through appropriate tailoring of the metallic nano-components and of the chemical composition of perovskite absorbers, one can also strengthen the optical plasmonic effect in perovskite cells, in addition to the electrical one. This demonstrates the great potential of the metallization of perovskite solar cells in order to increase their efficiency beyond the Shockley–Queisser limit. The most efficient of these involve bi-metallic multishell elongated nanoparticles (with double plasmon resonance) covered with a dielectric layer.

Metallization would not, however, change the market situation of perovskite cells in a decisive manner. The problem with these cells is different and consists in their poor durability and rapid degradation by oxygen and atmospheric water. Metallization can help in providing additional increase in efficiency but it is not a remedy for the obstacles associated with cell durability. However, in view of possible applications in space or on the Moon, perovskite cells that are capable of being printed in place from easy-to transport liquid precursors might provide a very good solution for energy sources for future extraterrestrial bases (perovskite should be more stable in space or on the Moon because of the absence of atmosphere there). In conventional terrestrial conditions, the usage of additional protection on perovskite cells is required in the form of encapsulation in glass or plastic, which limits the range of applications. The enhancement of cell efficiency via metallization may, however, increase this range. Moreover, in the case of perovskite cells, the metallization process is technically much easier in comparison to Si cells (in the latter case, special fixing of metallic particles on the upper surface of the Si cell is needed, which is not the case in perovskite cells). The cost of metallization is low–up to 1 USD per 1 m${}^{2}$ of the cell, because the nanoparticles are small and sparsely distributed (of the order of $10^{8-10}$ per cm${}^{2}$). This results in a low final cost, even using gold or silver particles. Depositioning of metallic particles in perovskite cells is also cheap and simple, especially if admixing particles for liquid precursors through the ink-jet printing of cell layers. 

## Figures and Tables

**Table 1:** Bandgaps in mixed-halide perovskites with large and small gaps (MA—CH${}_{3}$NH${}_{3}^{+}$, methylammonium) [44].

Composition	Bandgap (eV)
MAPb(I${}_{0.71}$Br${}_{0.29}$)${}_{3}$	1.71
MAPbI${}_{2}$Br	1.8
MAPb${}_{0.75}$Sn${}_{0.25}$(I${}_{0.4}$Br${}_{0.6}$)${}_{3}$	1.73
MASnI${}_{3}$	1.3
MAPb${}_{0.5}$Sn${}_{0.5}$I${}_{3}$	1.18

**Table 2:** Plasmon energies measured in metals.

Metal	Bulk pl [eV]	Surface pl in NPs (eV)
Li	6.6	3.4
Na	5.4	3.3
K	3.8	2.4
Mg	10.7	6.7
Al	15.1	8.8
Fe	10.3	5.0
Cu	6	3.5
Ag	3.8	3.5
Au	4.67	2.7

**Table 3:** Substrate material parameters ($m=9.1\times 10^{-31}$ kg, the mass of bare electron; lh–light holes, hh–heavy holes, L–longitudinal, T–transverse).

Semiconductor	$m_{n}^{*}$	$m_{p}^{*}$	$E_{g}$
Perovskite CH${}_{3}$NH${}_{3}$PbI${}_{3}$	1 m	1 m	1.6 eV
Si	0.9 m L[101], 0.19 m T[110]	0.16 m lh, 0.49 m hh	1.12 eV
GaAs	0.067 m	0.08 m lh, 0.45 m hh	1.35 eV
CIGS	0.09–0.13 m	0.72 m	1–1.7 eV

## Data Availability

Data available within the article.
